# The Dark Side of Resilience and Burnout: A Moderation-Mediation Model

**DOI:** 10.1371/journal.pone.0156279

**Published:** 2016-06-23

**Authors:** Luke Treglown, Kat Palaiou, Anthony Zarola, Adrian Furnham

**Affiliations:** 1 Research Department of Clinical, Educational and Health Psychology, University College London, London, United Kingdom; 2 Zeal Solutions, Nottingham, United Kingdom; 3 Norwegian Business School (BI), Nydalveien, Olso, Norway; University of Vienna, School of Psychology, AUSTRIA

## Abstract

This study tested whether specific dark-side traits may be beneficial in manifesting and maintaining Resilience, whilst others are vulnerability factors for Burnout. Four hundred and fifty-one (50 female) ambulance personnel completed three questionnaires as a part of a selection and development assessment. The study utilised the Hogan Development survey as a measure of dark side personality, the Copenhagen Burnout Inventory to assess work-related burnout, and the Resilience Scale– 14 to measure resilience levels. Those high on Excitable and Cautious but low on Bold and Reserved were linked to an increased vulnerability to Burnout. Also those high on Bold and Diligent yet low on the Excitable, Cautious, and Imaginative scales were more resilient. Structural Equation Modelling revealed that resilience plays both a mediating and moderating role on personality and burnout. Theoretical implications suggest future research assessing the predictive capacity of psychological variables on burnout should account the indirect effect of resilience.

## Introduction

Within the literature of Personality and Organisational Psychology, there is a growing interest in the comparing the explanatory power of ‘bright’ and ‘dark’ side personality traits. ‘Bright’ traits are those attributable to the interpersonal behaviours exhibited when we are being purposeful, positive, and at our best [1, 2]. Dark side traits, however, are those that have the potential to derail our personal and professional lives, and emerge with greater frequency when we let our guard down [3] We reveal our dark side when our cognitive resources to inhibit and suppress maladaptive impulses are depleted because we are stressed, tired, or overworked [4].

Taxonomies of dark personality traits attempt to characterise and define the interpersonally maladaptive behaviours that individuals engage in. That is, whilst dark personalities are extremely maladaptive, individuals are still able to survive (and in rare occasions flourish) in everyday society without impediment to their functioning [5].

One such account of dark personality is the Hogan Development Survey (HDS)[6]. The HDS measures 11 sub-clinical trait representations that incorporate the maladaptive interpersonal behaviours exhibited within the 11 personality disorders (detailed in Table 1). Conceptually, the 11 scales fit the Horney [7] three-tiered taxonomy of self-defeating behaviours: *Moving Away from Others* accounts for individuals who are intimidated by stress [8], and have a preference to withdraw and socially isolate themselves under stress; individuals with a predisposition for *Moving Towards Others* behaviours seek integration, and will become reliant on others to make decisions for them, renouncing responsibility and becoming submissive; and *Moving Against Others*, however, desire manipulation, detailing individuals who want to assert dominance and set boundaries when under stress, becoming aggressive at higher levels of these traits [9].

**Table 1:** Comparison in Themes Between DSM-IV-R Axis-II Personality Disorders and HDS ‘Dark Side’ Traits.

Axis-II Personality Disorder	Symptom Themes	HDS ‘Dark Side’ Trait	Trait Themes	Horney (1950) Classification
Borderline	Inappropriate anger; unstable and intense relationships alternating between idealization and devaluation.	Excitable	Moody and hard to please; intense, but short-lived enthusiasm for people, projects or things	‘Moving Away’
Paranoid	Distrustful and suspicious of others; motives are interpreted as malevolent.	Sceptical	Cynical, distrustful, and doubting other's true intentions	‘Moving Away’
Avoidant	Social inhibition; feelings of inadequacy and hypersensitivity to criticism or rejection	Cautious	Reluctant to take risks for fear of being rejected or negatively evaluated	‘Moving Away’
Schizoid	Emotional coldness and detachment from social relationships; indifferent to praise and criticism	Reserved	Aloof, detached, and uncommunicative; lacking interest in or awareness of the feelings of others	‘Moving Away’
Passive-Aggressive	Passive resistance to adequate social and occupational performance; irritated when asked to do something he/she does not want to	Leisurely	Independent; ignoring people's requests and becoming irritated or argumentative if they persist	‘Moving Away’
Narcissistic	Arrogant and haughty behaviours or attitudes; grandiose sense of self-importance and entitlement	Bold	Unusually self-confident; feelings of grandiosity and entitlement; overvaluation of one's capabilities	‘Moving Against’
Anti-Social	Disregard for the truth; impulsivity and failure to plan ahead; failure to conform with social norms	Mischievous	Enjoying risk taking and testing limits; needing excitement; manipulative, deceitful, cunning and exploitative	‘Moving Against’
Histrionic	Excessive emotionality and attention seeking; self-dramatizing, theatrical, and exaggerated emotional expression	Colourful	Expressive, animated, and dramatic; wanting to be noticed and needing to be the centre of attention	‘Moving Against’
Schizotypal	Odd beliefs or magical thinking; behaviour or speech that is odd, eccentric, or peculiar	Imaginative	Acting and thinking in creative and sometimes odd or unusual ways	‘Moving Against’
Obsessive-Compulsive	Preoccupations with orderliness, rules, perfectionism, and control; over conscientious and inflexible	Diligent	Meticulous, precise, and perfectionistic; inflexible about rules and procedures; critical of others' performance	‘Moving Towards’
Dependent	Difficulty making everyday decisions without excessive advise and reassurance; difficulty expressing disagreement out of fear of loss of support or approval	Dutiful	Eager to please and reliant on others for support and guidance; reluctant to take independent action or go against popular opinion	‘Moving Towards’

### Burnout and Resilience

In situations of prolonged interpersonal and emotional stress at work, employees can experience burnout [10]. Conversely, under immense stress some individuals demonstrate resilience and persevere in a relatively unwavering manner. Equally, can certain dark side traits be beneficial in stressful situations by providing additional psychological resources or aiding the adaptive allocation of these in a way that manifests resilience? By exploring the nature of these concepts, it becomes apparent that dark side personality has the potential to explain why these phenomena may be more likely to occur in certain individuals.

Firstly, burnout is conceptualised as an internal and emotional response to external stressors that consume, exceed, and deplete our personal and social resources [11]. Burnout represents the metaphorical incapacity for the fire within us to continue burning brightly [12]. The majority of research on burnout utilises a multidimensional account developed by Maslach and colleagues [13], which states burnout is comprised of feelings of emotional exhaustion, depersonalisation, and a lack of personal achievement [14]. However, within this field-dominating conceptualisation, burnout can only occur at work [13]; Maslach’s [15] burnout is thought to be context-free, referring specifically to a work-based phenomenon [12]. Kristensen et al. [16] argue that the three factors of Maslach’s burnout should be studied independently as the factors represent a mixture of coping strategies and end-states. Researchers have thus criticised the overuse of the Mashlach’s Burnout Inventory (MBI) in the literature as it creating a circular argument for burnout, with burnout now being what the MBI measures [16]. This study will instead view burnout singularly as fatigue and exhaustion resulting from chronic stress, focusing on this phenomenon within a work environment.

Resilience is a dynamic process [17], where an interaction occurs with the environment by negotiating and managing resources in response to the stressor [18]. Resilience emerges from ordinary processes that serve to protect the efficacy of these resource allocation systems. Recent researchers have argued that the focus of future resilience inquiry should attempt to identify factors that underlie these ordinary processes [19] in order to understand the protective roles they play.

The literature on resilience and burnout indicate that having a positive social network, good relationships with friends and colleagues, and having a supportive milieu are largely influential on the manifestation of both these phenomena. However, this research has focused upon ‘bright’ traits. What still is unknown is the role of dark personality; how do these traits, defined by behaviours that undermine interpersonal relationships, influence the phenomena that these social networks have been shown to benefit?

This study postulates that *Moving Away* characteristics will be common in both phenomena, being positively related to Burnout and negatively to Resilience. It is thought that these traits will be most detrimental due to the withdrawal and social isolation that underlies them; completely isolating yourself from social interaction and support will be more damaging compared to social interactions that are denoted by increase conflict and aggression (for instance, *Moving Against* traits).

Additionally, burnout and resilience will be distinguished by unique dark side traits. Resilience is hypothesised to be positively predicted by Diligent personality. Previous research has demonstrated that resilience is underlined by a drive and perseverance; ‘bright’ side analysis has revealed that having a highly conscientious personality is beneficial for resilience due to the task-orientated approach it elicits when dealing with stress [20, 21]. Diligence is an extension of these behaviours, and thus may be beneficial in manifesting resilience. Burnout, however, is hypothesised to be negatively predicted by Bold personality. The delusions of self-aggrandisement and over-evaluation of one’s capability will buffer against psychological exhaustion and fatigue. This differs from the role Diligence will play in resilience, as Bold personality will prevent burnout by reinforcing beliefs that one’s work is meaningful and of a good quality, rather than promoting an underlying tenacity to persevere.

Little research, however, has considered how resilience and burnout are related and interact. Models applied to medical students implicitly postulate both as outcome variables [22], suggesting that once our coping resources have run dry, we either burnout or exhibit resilience. It is unclear whether resilience plays a mediating or moderating role; does resilience moderate by impacting the strength of the personality-burnout relationship, or does it mediate by explaining the variance in the relationship between personality and burnout? Analyses of a structural model that depict these two relationships will offer a theoretical understanding of resilience’s role in burnout that has not been noted in the literature before.

## Method

### Participants

451 participants were assessed as a part of the selection and development of Ambulance Personnel who may be required to respond to high threat or terror incidences. Of these 401 were males and 50 were females. Their mean age was 39.9 years (*SD* = 8.33), with ages ranging from 21 to 64. University College London ethics committee approved the protocol prior to the study. Written and informed consent was provided by all participants was given before engaging in the study. The data was collected as a part of a larger consultation procedure for the specific organisation.

### Materials

#### Hogan Development Survey

The HDS [6] is made up of 154 items that address how the respondent interacts with their family, friends, and co-workers. The average coefficient alphas of .64 (ranging from .50 to .70), with an average test-retest reliability of .68, ranging from .58 (Leisurely) to .87 (Excitable) [23, 24].

#### Copenhagen Burnout Inventory

(CBI: 16) has three subscales: personal-, work-, and client-related burnout. For this study, the work-burnout dimension was utilised, focusing on the degree of fatigue and exhaustion that employees experience as a result of their work [25]. The work-related scale has reported reliability coefficients of .87 [26].

#### Resilience Scale– 14

(RS-14) [27] represents a shorter, 14-item version of the already well-established Resilience Scale (RS) [28], an inventory that has been deemed the ‘gold standard’ of resilience measurement within psychology [29]. The scale has demonstrated reliability coefficients ranging from .82 to .94 [30], with the scale being consistent in internal consistency, test-retest reliability, and concurrent validity to its longer version across cultures [31–33].

### Analyses

SPSS 22.0 was used to organise and clean the dataset, as well as being used to generate the correlations, regressions, and the Exploratory Factor Analysis (EFA) that appear within the results section. Descriptive statistics indicated that 127 participants were missing complete sets of either demographic, HDS, RS-14, or CBI data, and were thus excluded from the analysis. The literature specifies that the HDS forms three higher-order factors, so an EFA using Varimax rotation was performed in order to double-check the number of over-arching latent variables. Orthogonal rotation was chosen as this keeps the factors independent in rotation [34]. Varimax allows for a general approach to EFA that generates factors containing a smaller number of more highly loaded variables onto each factor, making more interpretable clusters [34].

In regards to Structural Equation Modelling (SEM), the Lavaan package [35] (version 0.5–20) in R (version 3.3.0) was used. SEM utilises a confirmatory approach in order to assess the structural interrelations and interactions between variables within the phenomenon, using theory to shape models that attempt to explain variance in the data. Maximum Likelihood was used for parameter estimation, as this has been deemed most appropriate for multivariate normal data and sample sizes are greater than 200 [36]. As there is no consensus within the literature as to which measure of goodness of fit is best, researchers have advised to use multiple tests [37]. The main indices that will be examined are RMSEA, where values of .08–0.05 represent adequate fit, and lower than .05 represent excellent fit [38]. Comparative fit index (CFI) was also used, where values greater than .95 are considered an excellent fit of the data [39] Finally, the Tucker-Lewis Index was assessed, where values over .90 are considered acceptable [40].

## Results

### Exploratory Factor Analysis

A Shapiro-Wilk test of normality found that resilience scores significantly differ from normal distribution (*p* < .001). Therefore, a Principal Axis Factoring method–the suggested method for non-normal data [41]–of extraction with orthogonal rotation (Varimax) was conducted on the 11 HDS traits in attempt to replicate and confirm the Horney [7] three-tier structure of dark side personality. Any factor loading below .30 was suppressed. The Kaiser-Meyer-Olkin measure of sample adequacy is over 0.5 (KMO = .734), indicating a significantly adequate sample. Bartlett’s test of spherecity was significant (χ^2^(55) = 1058.34, *p* < .001), indicating that the 11 HDS variables do not resemble an identity matrix and hold enough inter-item correlations for a sufficient EFA to have been conducted. Three components elicited eigenvalues that exceeded 1, which in combination explained 56.9% of the variance. The three-factor solution mimicked the Horney [7] model, with the factor 1 representing *Moving Against Others* (Bold, Mischievous, Imaginative, Colourful), factor 2 *Moving Away from Others* (Excitable, Sceptical, Cautious, Reserved, and Leisurely), and factor 3 *Moving Towards Others* (Dutiful and Diligent). Table 2 details the factor loadings of the HDS traits after the Varimax rotation.

**Table 2:** Principle Axis Factoring (with Varimax rotation) of the 11 HDS Scales.

	Component		
	1	2	3
Excitable		**.578**	
Sceptical	.407	**.464**	
Cautious		**.669**	
Reserved		**.505**	
Leisurely	.333	**.479**	
Bold	**.697**		
Mischievous	**.687**		
Colourful	**.623**		
Imaginative	**.552**		
Diligent			**.616**
Dutiful			**.367**

A Shapiro-Wilk test of normality found that resilience scores significantly differ from normal distribution (*p* < .001). Therefore, a Principal Axis Factoring method of extraction with Oblimin rotation (Direct Oblimin) was also conducted on the 14 items of the RS-14. This was done primarily to reduce the number of observed variables comprising Resilience within SEM. As before, any factor loading that fell below .30 was supressed. The Kaiser-Meyer-Olkin measure of sample adequacy was noted to be over 0.5 (KMO = .909). Furthermore, Bartlett’s test of spherecity was also found to be significant (χ^2^(91) = 1820.51, *p* < .001. This three-factor solution, however, does not structurally replicate what has been noted previously in the literature. The results of the factor analysis can be seen in Table 3.

**Table 3:** Principle Axis Factoring (with Direct Oblimin Rotation) of the 14 RS-14 items.

	Component		
	1	2	3
1 –I feel proud that I have accomplished things in life	**.455**		
2 –I keep interested in things			**-.536**
3 –My life has meaning	**.514**		
4 –I am determined	**.372**		-.328
5 –I have self-discipline	**.344**		
6 –I usually take things in my stride		**-.367**	
7 –I can usually find something to laugh about	**.440**	-.335	
8 –I usually manage, one way or another		**-.757**	
9 –I feel that I can handle many things at a time		**-.652**	
10 –I can get through difficult times because I have experienced difficulty before	**.365**		
11 –In an emergency, I am someone people can generally rely on	**.498**		
12 –When I’m in a difficult situation, I can usually find a way out of it			
13 –I am friends with myself			**-.600**
14 –My belief in myself gets me through hard times	**.400**		

### Burnout: Correlations and Regressions

Table 4 shows the correlation results for burnout and the dark side traits. Overall, seven of the 11 HDS variables significantly correlate with Burnout. Six significantly positively correlated with burnout scores: Excitable Sceptical, Cautious, Reserved, Leisurely and Imaginative. However, one trait significantly negatively correlated with burnout, namely Bold. *Moving Away from Others* was the only higher order factor to significantly correlate with Burnout, showing a strong positive relationship (*r* = .41; *p* < .001). Neither *Moving Against Others* nor *Moving Towards Others* significantly correlated with burnout.

**Table 4:** Correlations Between CBI work-related Burnout and the 11 HDS Dark Side Traits.

	Burnout	Excitable	Sceptical	Cautious	Reserved	Leisurely	Bold	Mischievous	Colourful	Imaginative	Diligent	Dutiful
Burnout	1											
Excitable	.41**	1										
Sceptical	.22**	.28**	1									
Cautious	.39**	.45**	.23**	1								
Reserved	.11*	.29**	.28**	.26**	1							
Leisurely	.24**	.22**	.41**	.36**	.21**	1						
Bold	-.10*	-.06	.28**	-.21**	-.07	.23**	1					
Mischievous	.06	.03	.31**	-.15**	.06	.25**	.39**	1				
Colourful	-.06	-.12**	.07	-.31**	-.19**	.09	.47**	.43**	1			
Imaginative	.14**	.13**	.30**	.03	.11*	.28**	.37**	.40**	.29**	1		
Diligent	.01	.03	.26**	.10*	-.05	.23**	.30**	.03	-.03	.16**	1	
Dutiful	.07	.12**	.02	.22**	-.13**	.14**	-.02	-.12*	-.06	-.03	.19**	1

Correlations with ** are significant p < .001;

* are significant p < .05.

A two-step hierarchical regression was conducted in order to investigate which of the three higher factors of HDS predict burnout. In the first step we inserted age and gender and in the second the three factors. The analysis revealed *Moving Away* was the only predictor for burnout. Results can be seen in Table 5.

**Table 5:** Regression of Age, Gender, and three higher-order HDS Dark Side facets as predictors of CBI work-related Burnout.

		Burnout	
		*β*	t
Step 1	Age	-.030	-.641
	Gender	.043	.905
*F-Score*	*F(2*, *448) = .703*		
*R*^*2*^	*.003*		
*ΔR*^*2*^	*-.001*		
Step 2	Age	-.077	-1.74
	Gender	.028	.635
	*Moving Away*	.431	9.72**
	*Moving Against*	-.053	-1.21
	*Moving Towards*	-.037	-.832
*F-Score*	*F(5*, 450) = 19.52**		
*R*^*2*^	*.180*		
*ΔR*^*2*^	*.171*		

Note:

** = *p* < .01

A further two step hierarchical regression was conducted in order to determine specifically which of the 11 traits of HDS were predictive of burnout After controlling for age and gender, accounted for an additional 24.2% of the variance, with Excitable and Cautious positively predicting burnout, whilst Bold and Reserved negatively predicting. Age became a significant predictor in the model with the second step. Results can be seen in Table 6.

**Table 6:** Regression of Age, Gender, and HDS Dark Side as predictors of CBI work-related Burnout.

		Burnout	
		*β*	t
Step 1	Age	-.030	-.641
	Gender	.043	.905
*F-Score*	*F(2*, *448) = .703*		
*R*^*2*^	*.003*		
*ΔR*^*2*^	*-.001*		
Step 2	Age	-.09	-2.03*
	Gender	-.01	-.237
	Excitable	.272	5.66**
	Sceptical	.087	1.72
	Cautious	.249	4.66**
	Reserved	-.096	-2.06*
	Leisurely	.083	1.64
	Bold	-.144	-2.67**
	Mischievous	.062	1.20
	Colourful	.014	.262
	Imaginative	.076	1.57
	Diligent	-.033	-.707
	Dutiful	-.038	-.854
*F-Score*	*F(13*, *437) = 12*.06**		
*R*^*2*^	.*264*		
*ΔR*^*2*^	.*242*		

Note:

** = *p* < .01;

* = *p* < .05

### Resilience: Correlations and Regressions

Table 7 shows the results for the correlations between Resilience and the 11 HDS traits. Similarly to Burnout, seven of the 11 traits correlate significantly with resilience. Four traits significantly positively correlated with resilience: Bold, Diligent, Colourful, and Imaginative. The other three traits were found to significantly negatively correlate: Cautious, Excitable, and Reserved. Furthermore, looking at the three-factor model of the HDS scale, demonstrating that *Moving Away* significantly negatively correlated (*r* = -.29), whilst *Moving Against* significantly positively correlated (*r* = .17), with resilience scores. *Moving Towards* was not found to significantly correlate with resilience.

**Table 7:** Correlations Between Resilience (RS-14) and the 11 HDS Dark Side Traits.

	Resilience	Excitable	Sceptical	Cautious	Reserved	Leisurely	Bold	Mischievous	Colourful	Imaginative	Diligent	Dutiful
Resilience	1											
Excitable	-.30**	1										
Sceptical	-.08	.28**	1									
Cautious	-.35**	.45**	.27**	1								
Reserved	-.19**	.29**	.28**	.26**	1							
Leisurely	-.08	.22**	.42**	.36**	.21**	1						
Bold	.23**	-.06**	.28**	-.21**	-.07	.23**	1					
Mischievous	.03	.03	.31**	-.15**	.06	.25**	.39**	1				
Colourful	.12*	-.12**	.07	-.31**	-.19**	.09	.47**	.43**	1			
Imaginative	.10*	.13**	.30**	.03	.11*	.28**	.37**	.40**	.29**	1		
Diligent	.15**	.03	.26**	.10*	-.05	.23**	.30**	.032	-.01	.16**	1	
Dutiful	-.04	.12**	.02	.22**	-.13**	.14**	-.02	-.12*	-.06	-.03	.19**	1

Correlations with ** are significant p < .001;

* are significant p < .05.

Table 8 shows the results of the two-step hierarchical regression using the three higher order factors as predictors for resilience. After controlling for age and gender, the three higher-order factors accounted for 13.9% of the variance. All three factors were found to significantly predict Resilience, with *Moving Away* negatively predicting resilience whilst *Moving Against* and *Moving Towards* positively predicting. As with burnout, further hierarchical regressions were conducted to see specifically which of the 11 dark side traits significantly predict Resilience.

**Table 8:** Regression of Age, Gender, and three higher-order HDS Dark Side facets as predictors of Resilience (RS-14).

		Resilience	
		*β*	t
Step 1	Age	-.030	-.641
	Gender	.043	.905
*F-Score*	*F(2*, *448) =* .*703*		
*R*^*2*^	.*003*		
*ΔR*^*2*^	*-*.*001*		
Step 2	Age	.020	.440
	Gender	.081	1.84
	*Moving Away*	-.344	-7.63**
	*Moving Against*	.199	4.45**
	*Moving Towards*	.138	3.09**
*F-Score*	*F(5*, *450) = 15*.51**		
*R*^*2*^	.*148*		
*ΔR*^*2*^	.*139*		

Note:

** = *p* < .01

Table 9 shows the results of the final two -step hierarchical regression. After age and gender were accounted for, the 11 HDS traits accounted for an additional 20.3% of the variance, with Diligent, Bold and Imaginative positively predicting resilience, whilst Excitable (and Cautious negatively predicting.

**Table 9:** Regression of Age, Gender, and HDS Dark Side as predictors of Resilience (RS-14).

		Resilience	
		*β*	t
Step 1	Age	-.050	-1.05
	Gender	.058	1.22
*F-Score*	*F(2*, *448) = 1*.*49*		
*R*^*2*^	.*007*		
*ΔR*^*2*^	.*002*		
Step 2	Age	.02	.364
	Gender	.11	2.52*
	Excitable	-.160	-3.25**
	Sceptical	-.040	-.780
	Cautious	-.286	-5.21**
	Reserved	-.064	-1.35
	Leisurely	.017	.331
	Bold	.162	2.93*
	Mischievous	-.085	-1.61
	Colourful	-.062	-1.14
	Imaginative	.118	2.38*
	Diligent	.122	2.60*
	Dutiful	.007	.157
*F-Score*	*F(13*, *437) = 9*.83**		
*R*^*2*^	.*226*		
*ΔR*^*2*^	.*203*		

Note:

** = *p* < .01;

* = *p* < .05

### Structural Model

SEM was used to analyse a moderation and mediation model in order to explore how an individual’s level of resilience can impact the relationship between burnout and dark side personality, represented by the 11 HDS dark side traits. The model analysed whether this relationship is mediated by Resilience—that is, can the impact of personality on burnout be contingent upon and filtered by the individual’s level of resilience–or whether this relationship instead is moderated by Resilience—burnout occurs as an interaction between dark personality and resilience.

For the model, Resilience and Work Burnout were entered as the latent variables. Due to the unavailability of the item-level data, the 11 dark side personality traits were treated as observed variables. Resilience was represented by three observed variables, representing mean scores for each of the three factors that were generated via an EFA on the RS-14. This latent variable depicts an individual’s general capacity for resilience. A similar process was attempted for CBI work-related burnout, but only one factor emerged. Therefore, the six items of the CBI work-related burnout scale represented the latent variable of Work Burnout. This variable represents an individual’s propensity to feel physically and emotionally drained as a result of their working conditions and environment. Eleven moderation terms were also entered, representing the interaction between each individual HDS trait and Resilience. The variables that were used in interaction terms were mean centred (the variable mean is subtracted from all observations) before multiplication, as this has been shown to create orthogonal interaction terms that are appropriate for regression and SEM [42]. These eleven interaction terms were regressed onto Work Burnout to test the moderating role of Resilience. Non-significant regressions were removed in a step-wise fashion, where the model was re-tested until only significant terms remained. HDS traits Sceptical, Leisurely, Colorful, and Dutiful were completely removed from the model based on being non-significant predictors of both Resilience and Work Burnout. All moderation terms apart from Diligent*Resilience where non-significant, and thus removed from the model.

**Fig 1:**
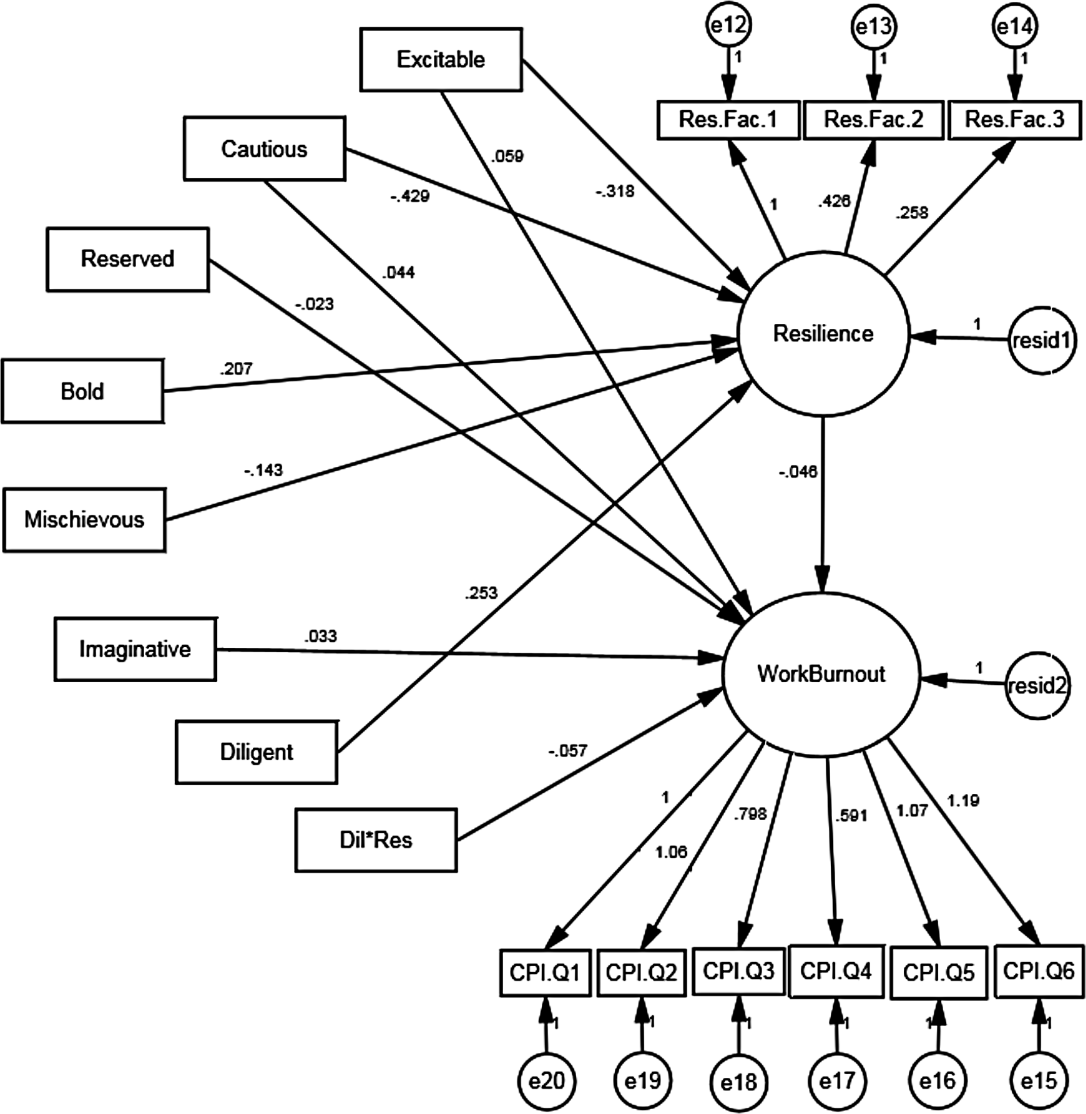
Structural Equation Model testing the mediating role of Resilience on the relationship between ‘dark’ side personality and Work Burnout.

The results of this model are shown in Fig 1. The mediating model yielded a significant chi-square statistic (χ^2^(87) = 189.99, *p* < .001), indicating that the model deviates from the structure of the data. However, researchers have indicated that large sample sizes artificially inflate chi-square values, causing a rejection of the model [43]. For this reason, other absolute fit indices were utilised, revealing a good fit of the model: CFI = .92; TLI = .90; RMSEA = .051 (90% CI Upper Limit = .041; 90% CI Lower Limit = .061).

As expected, Excitable and Cautious negatively directly impacted Resilience, whilst Bold and Diligence had a positive direct impact. Differing from the regression models, Imaginative was no longer a significant predictor of Resilience, whilst Mischievous became significant.

Excitable, Cautious, and Imaginative were found to have a significantly positive direct impact on Work Burnout. Reserved and the moderating term Diligence*Resilience were found to negatively impact Work Burnout. As anticipated, Resilience negatively explains variance in Work Burnout. Bold was no longer a significant predictor of Work Burnout when Resilience was entered into the model.

The mediating role of Resilience was investigated by assessing the indirect impact dark side personality would have on Work Burnout. Researchers have suggested that to test the significance of indirect effects, bootstrapping procedures should be used [44], thus 1,000 bootstrap samples were created (as recommended by Cheung & Lau [45]. Using the bias-corrected percentile method, a significant indirect effect was noted for Bold (β = -.01; *p* = .011). These results indicate that Resilience plays an additional role by fully mediating the impact of Bold, whilst moderating the impact of Diligence, on Work Burnout.

## Discussion

In this study it was found that moody, emotionally volatile, Excitable personalities were at greater risk for Burnout. Excitable personalities have short-lived enthusiasm for projects and people, as well as a heightened sensitivity to betrayal, putting them at risk due to their increased likelihood to actively cut ties with their friends and colleagues. The Cautious personality was also associated with a higher risk of burnout. Cautious personalities are denoted by a social inhibition and social risk-aversion due to the fear of being rejected, reducing the number of positive social interactions the individual engages in.

Dark personality was also hypothesised to impact burnout in a positive way; certain maladaptive traits would potentially buffer against the onset of burnout. Bold personality, characterised by self-confidence, a sense of entitlement, and an over-evaluation of one’s capability, was postulated to buffer against burnout due to a less frequent perceptions of low self-worth and doubts about the quality of work that can be delivered. This hypothesis was confirmed, but *Moving Against* as a whole was not a positive predictor of burnout, implying there are beneficial properties of bold personality that do not underlie or occur within all *Moving Against* traits.

Furthermore, burnout was also negatively predicted by the dark side trait Reserved. This result is surprising, as reserved personalities are socially aloof, detached, and uncommunicative; they lack interest in or an awareness of the feelings of others. This lack of interest in social attachment could act as a social buffer; adaptive (not taking things personally) over dysfunctional (withdrawing when someone gets too close) interpersonal detachment has been shown to reduce anxiety related to daily stressors [46]. However, this does not provide an active benefit through resilience, but merely prevents burnout.

It is worth considering that the avoidant coping styles that are seen in Reserved personality are common strategies that individuals use to alleviate stress [47]. In turn, these avoidant styles have been connected with depersonalisation, which when combined with emotional exhaustion and a lack of personal achievement represents Maslach’s multi-dimensional view of burnout [15, 48]. However, the conceptualisation of burnout within this study was one-dimensional, focusing on feelings of fatigue and exhaustion alone as the core of burnout [16].

### What does dark side personality tell us about Resilience?

As with burnout, the socially damaging properties seen within *Moving Away* traits were hypothesised to be additionally detrimental to the well-being of employees by undermining their capacity to exhibit resilience. This hypothesis was confirmed. As with burnout, resilience was specifically negatively predicted by excitable and cautious personality types. It appears that the behavioural tendencies of emotional volatility or social inhibition, as seen within excitable and cautious respectively, not only increase the propensity to burnout, but also undermine the capacity of resilience.

It was also hypothesised that resilience would be benefited by certain dark side traits, as they may inadvertently provide the individual with additional resources to cope at the potential expense of healthy relationships. The Bold personality was found to be a positive predictor of resilience. One such example is the trait ‘self-enhancement’, which is defined as a disposition to be overly positive towards the self or possessing unrealistic self-serving biases [49–52].

Trait self-enhancement has been viewed as a double-edged sword; higher levels of self-enhancement have been noted in resilient individuals [52], but these individuals have also been viewed by family and peers as being less honest and socially adjusted [49]. Despite these negative impressions, self-enhancers have been noted to view their milieu as supportive towards them maintaining a positive adjustment. In a certain capacity, it appears that bold individuals are immune to the negative opinions and views of others [53] and reinterpret their presence in a positive and supporting light. The overall impact of Bold personality may benefit resilience by providing additional protective resources through a combined effort of personal self-confidence, focusing on the positives and ignoring the negatives both personally and socially, and blaming others for their own failings.

Additionally, both Diligent and Imaginative personality styles were positive predictors of resilience. This result supports previous research that states resilience is underpinned by high conscientiousness [20, 21], as this manifests an active and task-orientated approach to dealing with stress, similar to diligent personality. Imaginative personality, however, is characterised by thoughts and behaviour that are considered creative, yet odd and unusual. Traits associated with Imaginative personality have previously been shown to strongly correlate with creativity [54].

### What does dark side personality tell us about how Resilience and Burnout interact?

The literature offers no foundations on whether resilience would act in a mediating or moderating capacity, so a model was generated in order to ascertain how and where Resilience interacts with personality and Burnout. SEM analysis revealed that resilience moderated the relationship between personality and burnout; the interaction term of Diligence and Resilience was found to be a significant negative predictor of burnout.

Resilience was also found to be a mediator between certain dark personality traits and burnout. Initial regressions indicated that the HDS trait Bold was a negative predictor of Work Burnout. However, when Resilience was also included in the model, Bold was no longer a significant predictor. Analysis of the indirect effects found that Resilience fully mediated the relationship between Bold and Work Burnout.

The success of this model therefore implies that Resilience acts as both a mediating and moderating filter for different dark side traits. Dark side traits regulate the extent to which an individual can act resiliently, with high Bold providing the optimal level. In turn, this manifested resilience capacity influences the potential of burnout development, with a greater capacity reducing its likelihood. Furthermore, the effect of Diligence reducing an individual’s risk of burnout is contingent on high levels of resilience. High conscientiousness, therefore, only helps prevent work burnout when the individual also possesses a higher level of coping resources.

However, this study is not without limitations. Firstly, the demographics of the sample are overly androcentric and industry-specific, with only ambulance personnel being assessed and males constituting roughly 90% of the participants. Despite this, the sample does accurately represent the gender proportionality within the paramedic service. Adriaenssens et al. [55] noted that females represent a far larger segment of in-hospital emergency services than ambulance-related services. Future research needs to assess the dark side of resilience and burnout in a more general population, as well as looking at other industries.

Furthermore, a factor analysis of the RS-14 did not fit with the structures that have been noted in previous literature. Wagnild [20] noted that the RS-14 was best represented by a single-factor solution, a result that has been replicated cross-culturally in Brazilian [56] and Portuguese [57, 58] samples. Future research, therefore, needs to focus on further examining the factor structure of the RS-14, potentially aligning with previous researchers in considering the removal of items that hold multiple loadings or ambiguous amounts of additional variance [56].

## References

[1] Hogan R, Kaiser RB. What we know about leadership. Rev Gen Psychol, 2005 6;9(2):169–180.

[2] Kaiser RB, LeBreton JM, Hogan J. The dark side of personality and extreme leader behavior. Appl. Psychol. 2015 1 1;64(1):55–92.

[3] Harms PD, Spain SM. Beyond the bright side: Dark personality at work. Appl. Psychol. 2015 1 1;64(1):15–24.

[4] Hogan R, Hogan J. Assessing leadership: A view from the dark side. Int J Select Assess. 2001 3 1;9(1‐2):40–51.

[5] Paulhus DL. Toward a taxonomy of dark personalities. Curr. Dir. Psychol. 2014 12 1;23(6):421–6.

[6] Hogan R. Hogan development survey manual Tulsa, OK: Hogan Assessment Systems; 1997.

[7] Homey K. Neurosis and human growth New York: W W Norton and Company; 1997.

[8] Hogan J, Hogan R, Kaiser RB. Management derailment. APA handbook of industrial and organizational psychology. 2010 7;3:555–75.

[9] De Fruyt F, Wille B, Furnham A. Assessing aberrant personality in managerial coaching: Measurement issues and prevalence rates across employment sectors. Eur. J. Pers. 2013 11 1;27(6):555–64.

[10] Schaufeli WB, Leiler P. Maslach Burnout Inventory-General Survey (MBI-GS). 3rd ed. Maslach C. MBI Manual; 1996.

[11] Maslach C, Leiter M. P. The truth about burnout. San Francisco: Jossey-Bass; 1997.

[12] Schaufeli WB, Leiter MP, Maslach C. Burnout: 35 years of research and practice. Career Dev. Int. 2009 6 19;14(3):204–20.

[13] Bianchi R, Truchot D, Laurent E, Brisson R, Schonfeld IS. Is burnout solely job‐related? A critical comment. Scand. J. Psychol. 2014 Aug 1;55(4):357–61. 10.1111/sjop.12119 24749783

[14] Demerouti E, Mostert K, Bakker AB. Burnout and work engagement: a thorough investigation of the independency of both constructs. J. Occup. Health. Psychol. 2010 7;15(3):209 10.1037/a0019408 20604629

[15] Maslach C. A multidimensional theory of burnout. Theory Org, Stress. 1998 10 29;68.

[16] Kristensen TS, Borritz M, Villadsen E, Christensen KB. The Copenhagen Burnout Inventory: A new tool for the assessment of burnout. Work Stress. 2005 7 1;19(3):192–207.

[17] Masten AS. Ordinary magic: Resilience processes in development. Am. Psychol. 2001 3;56(3):227 11315249 10.1037//0003-066x.56.3.227

[18] Windle G. Psychological resilience as a resource for later life. InGerontologist 2011 Nov 1 (Vol. 51, pp. 331–331). JOURNALS DEPT, 2001 EVANS RD, CARY, NC 27513 USA: OXFORD UNIV PRESS INC.

[19] Winwood PC, Colon R, McEwen K. A practical measure of workplace resilience: Developing the resilience at work scale. J. Occup. Env. Med. 2013 10 1;55(10):1205–12. 24064782 10.1097/JOM.0b013e3182a2a60a

[20] Wagnild G. A review of the Resilience Scale. J Nurs Meas. 2009 8 1;17(2):105–13. 19711709 10.1891/1061-3749.17.2.105

[21] Campbell-Sills L, Cohan SL, Stein MB. Relationship of resilience to personality, coping, and psychiatric symptoms in young adults. Behav Res Ther. 2006 4 30;44(4):585–99. 15998508 10.1016/j.brat.2005.05.001

[22] Dunn LB, Iglewicz A, Moutier C. A conceptual model of medical student well-being: promoting resilience and preventing burnout. Acad. Psychiat. 2008 1 1;32(1):44–53. 10.1176/appi.ap.32.1.44 18270280

[23] Fico J., Hogan R., Hogan J. Interpersonal compass manual and interpretation guide. Tulsa, OK: Hogan Assessment System; 2000.

[24] Fox G., Huebner E. S. Review of the Hogan development survey Mental measurements yearbook. 14 ed. Lincoln, NE: The University of Nebraska Press; 2001.

[25] Avanzi L, Zaniboni S, Balducci C, Fraccaroli F. The relation between overcommitment and burnout: does it depend on employee job satisfaction?. Anxiety Stress Coping. 2014 7 4;27(4):455–65. 10.1080/10615806.2013.866230 24245551

[26] Borritz M., Kristensen T. S. Copenhagen Burnout Inventory: Normative data from a representative Danish population on Personal Burnout and results from the PUMA study on Personal Burnout, Work Burnout, and Client Burnout. Copenhagen: National Institute of Occupational Health; 2001.

[27] Wagnild GM. The resilience scale user's guide: For the US English version of the Resilience Scale and the 14-item Resilience Scale (RS-14). Guinn PE, editor. Resilience center; 2011.

[28] Wagnild G, Young H. Development and psychometric evaluation of the Resilience Scale. J Nurs Meas. 1993;1:165–78. 7850498

[29] Ahern NR, Kiehl EM, Lou Sole M, Byers J. A review of instruments measuring resilience. Issues Compr Pediatr Nurs. 2006 1 1;29(2):103–25. 16772239 10.1080/01460860600677643

[30] Pritzker S, Minter A. Measuring adolescent resilience: An examination of the cross-ethnic validity of the RS-14. Child Youth Serv Rev. 2014 9 30;44:328–33.

[31] Nishi D, Uehara R, Kondo M, Matsuoka Y. Reliability and validity of the Japanese version of the Resilience Scale and its short version. BMC Res Notes. 2010 11 17;3(1):310 10.1186/1756-0500-3-310 21083895 PMC2993730

[32] Abiola T, Udofia O. Psychometric assessment of the Wagnild and Young's resilience scale in Kano, Nigeria. BMC Res Notes. 2011 11 23;4(1):509. 22112503 10.1186/1756-0500-4-509 PMC3261834

[33] Girtler N, Casari EF, Brugnolo A, Cutolo M, Dessi B, Guasco S, et al Italian validation of the Wagnild and Young Resilience Scale: a perspective to rheumatic diseases. Clin Exp Rheumatol. 2009 12;28(5):669–78. 20822709

[34] Field A. Discovering statistics using SPSS. London: Sage publications; 2009.

[35] Rosseel Y. lavaan: An R package for structural equation modeling. Journal of Statistical Software. 2012 5 24;48(2):1–36.

[36] Hox JJ, Bechger TM. An introduction to structural equation modelling. Family Science Review. 1998;11: 354–373

[37] Kline RB. Principles and practice of structural equation modelling. New York: Guilford Press; 1998.

[38] MacCallum RC, Browne MW, Sugawara HM. Power analysis and determination of sample size for covariance structure modeling. Psychol. methods. 1996 6;1(2):130–149.

[39] Hooper D, Coughlan J, Mullen M. Structural equation modelling: Guidelines for determining model fit. EJBRM. 2008:6; 53–60.

[40] Hu LT, Bentler PM. Evaluating model fit In Hoyle RH (Ed.), Structural equation modeling: Concepts, issues, and applications. Thousand Oaks, CA: Sage; 1998 p. 76–99.

[41] Costello AB, Osborne JW. Best Practices in Exploratory Factor Analysis: Four Recommendations for Getting the Most From Your Analysis. PARE. 2005;10(7):1–9.

[42] Little TD, Card NA, Bovaird JA, Preacher KJ, Crandall CS. Structural equation modeling of mediation and moderation with contextual factors In: Little TD, Card NA, editors. Modelling contextual effects in longitudinal studies. Abingdon-on-Thames: Routledge; 2007 pp. 207–30.

[43] Bentler PM, Bonett DG. Significance tests and goodness of fit in the analysis of covariance structures. Psychol. Bull. 1980 11;88(3):588–606.

[44] Shrout PE, Bolger N. Mediation in experimental and nonexperimental studies: new procedures and recommendations. Psychol. Methods. 2002 12;7(4):422–45. 12530702

[45] Cheung GW, Lau RS. Testing mediation and suppression effects of latent variables: Bootstrapping with structural equation models. Organ. Res. Meth. 2007 7 23 10.1177/1094428107300343

[46] Denckla CA, Bornstein RF. Toward a more nuanced conceptualization of interpersonal distancing: Differential relationships of adaptive and dysfunctional detachment to stress-based anxiety in college students. Pers Indiv Differ. 2015 8 31;82:148–52. 10.1016/j.paid.2015.03.008 PMC6395042 30828122

[47] Carver CS, Scheier MF, Weintraub JK. Assessing coping strategies: a theoretically based approach. J Pers Soc Psychol. 1989 2;56(2):267–83. 2926629 10.1037//0022-3514.56.2.267

[48] Thoresen CJ, Kaplan SA, Barsky AP, Warren CR, de Chermont K. The affective underpinnings of job perceptions and attitudes: a meta-analytic review and integration. Psychol. Bull. 2003;129(6): 914–945. 14599288 10.1037/0033-2909.129.6.914

[49] Bonanno GA, Rennicke C, Dekel S. Self-enhancement among high-exposure survivors of the September 11th terrorist attack: Resilience or social maladjustment?. J Pers Soc Psychol. 2005 6;88(6):984–98. 15982117 10.1037/0022-3514.88.6.984

[50] Bonanno GA. Loss, trauma, and human resilience: have we underestimated the human capacity to thrive after extremely aversive events?. Am Psychol. 2004 1;59(1):101–03. 10.1037/0003-066X.59.1.20 14736317

[51] Hasui C, Igarashi H, Shikai N, Shono M, Nagata T, Kitamura T. The resilience scale: A duplication study in Japan. OFSJ. 2009;2:15–22.

[52] Gupta S, Bonanno GA. Trait self-enhancement as a buffer against potentially traumatic events: A prospective study. Psychol Trauma. 2010 6;2(2):83–92.

[53] Robins RW, Beer JS. Positive illusions about the self: short-term benefits and long-term costs. J Pers Soc Psychol. 2001 2;80(2):340–52. 11220450 10.1037/0022-3514.80.2.340

[54] Batey M, Furnham A. The relationship between measures of creativity and schizotypy. Pers Indiv Diff. 2008 12 31;45(8):816–21.

[55] Adriaenssens J, De Gucht V, Maes S. Determinants and prevalence of burnout in emergency nurses: A systematic review of 25 years of research. Int J Nurs Stud. 2015 2 28;52(2):649–61. 10.1016/j.ijnurstu.2014.11.004 25468279

[56] Damásio BF, Borsa JC, da Silva JP. 14-item resilience scale (RS-14): psychometric properties of the Brazilian version. J Nurs Meas. 2011 12 1;19(3):131–45. 22372090 10.1891/1061-3749.19.3.131

[57] Oliveira A, Matos AP, do Rosário Pinheiro M, Oliveira S. Confirmatory Factor Analysis of the Resilience Scale Short form in a Portuguese Adolescent Sample. Procedia Soc. Behav. Sci. 2015 1 6;165:260–6.

[58] Pinheiro MR, Matos AP. Exploring the Construct Validity of the Two Versions of the Resilience Scale in an Portuguese Adolescent Sample. EJSBS. 2013;2(10):178–89.

